# Acting as a Mental Health Expert by Experience and Its Impact on Social Identity

**DOI:** 10.1007/s10597-023-01207-w

**Published:** 2023-11-17

**Authors:** Kari Soronen

**Affiliations:** Faculty of Social Sciences, University of Lapland, Rovaniemi, Finland

**Keywords:** Experts by experience, Mental health services, Social identity, Finland

## Abstract

This study was conducted to examine the construction of social identity among mental health experts by experience working in Finnish municipal mental healthcare services. The construction of social identity is approached as an ongoing lifelong process that is significantly affected by lived experiences with mental health problems and recovery from them. The research data consist of focus group discussions, and the analysis is based on a thematic design that is initially material-driven. As a result, four categories are formed to describe the effect of acting as an expert by experience on social identity and the importance of the issue. Experts by experience have to consider profound questions about their identity and future in their new life situation. The individual meanings of acting as an expert by experience are considered particularly important. The support provided by group members builds confidence in one’s chances of success and thereby supports the development of social identity. Social identities of experts by experience are partially built in encounters with professionals representing the mental health care system. This creates opportunities for new roles for those who act as experts by experience.

## Introduction

In describing one’s own life, people define themselves in relation to other people and the surrounding community (Prince et al., 2017). In this study, I examine the construction of social identity among mental health experts by experience working in Finnish municipal mental healthcare services. Internationally, experiential expertise has become increasingly important in developing high-quality mental health services (Mancini, 2018; Nossek et al., 2021; Glasby & Beresford, 2006). The input of experts by experience is considered to enable a more patient- and client-centered approach and to result in a better quality of mental health services (Kaminskiy et al., 2013; Martin & Finn, 2011). Through experiential expertise, experts can bring considerable know-how of psychiatric rehabilitation and recovery orientation to service encounters (Leamy et al., 2011; van Weeghel et al., 2019), thereby emphasizing the dignity and relevance of individual experiences.

Experiential expertise includes many dimensions, of which peer support is one important part (e.g., Faulkner, 2017). Studies worldwide have focused on working as peers in mental health services. In Finland, peer support is generally separated from activities related to participation in the planning, development, and evaluation of mental health work structures when working as paid experts by experience. The division between the peer support of experts by experience and participation work tasks related to the development of broader mental health work remains debatable (Meriluoto, 2018b). So far, in Finland, there is no consensus on the content of the activities of experts by experience. In my research, I use the concepts of mental health expert by experience and experiential expertise, which are generally used in Finland. By contrast, in English-language research literature, the concept of peer is mainly used (e.g., peer support, peer support worker) (Davidson et al., 2012; Puschner et al., 2019).

Experts by experience are playing an increasingly recognized role working alongside mental health professionals in municipal mental health work (Lehtinen & Taipale, 2001; Jones & Pietilä, 2020).

Experts by experience are not permanent staff, and they are not required to have professional training in mental health work. They often work on fixed-term or part-time salaried contracts. In addition, in Finland, an expert by experience can work without an employment contract or salary. In addition to peer support work, their duties also include other tasks, such as guiding therapeutic groups with mental health professionals and participating in development and training tasks. Therefore, working as a paid expert by experience has a broader role than working as a paid peer support worker, which are also used alongside services. Another significant difference is that the hiring of experts by experience aims at a broader political change towards services where customer participation is at the core of services and their development (e.g., Lindström & Rantanen, 2021).

They are also playing an important role in nongovernmental mental health organizations (Meriluoto, 2018a). In Finland, this development has been strongly supported through political measures, and the role of experts by experience has been recognized in various national recommendations (Mieli 2009 Working Group & Ministry of Social Affairs and Health, 2010). In Finland, legitimizing the work of experts by experience has been seen as a way to diminish clients’ marginalization and the lack of their legitimacy in service systems as well as to influence problems, including the high cost, fragmentation, and inefficiency, of welfare services (e.g., Meriluoto, 2018b). Previous studies have shown that the work of experts by experience has positive effects on the life management, well-being, and life satisfaction of people suffering from mental health challenges (Cook et al., 2010; Davidson et al., 2012; Shalaby & Agyapong, 2020).

Internationally, studies of experiential expertise have focused on recognizing the knowledge of experts by experience when they work as peers in mental health services (Davidson et al., 2012; Puschner et al., 2019). Goldberg et al. (2015) used the concept of “therapatient” when describing the process of integration between one’s own mental health challenges experienced as a patient and professional knowledge. Therapatient refers to professionals who are, or have earlier been also patients. The therapatient concept can be seen as an effort to assimilate experiential expertise into mental health work. Own experiences, expertise, and professionalism can be combined in the same person (Beresford & Boxall, 2015; Rissanen, 2015). A mental health expert by experience commonly refers to a person who has formerly experienced mental health disorders and used mental health services and recovered (Curran et al., 2015; Rissanen, 2015). The notion of “recovered” embodies the possibility of living a satisfying life despite the limitations caused by the mental health challenges (Anthony, 1993). Recovering does not mean the complete end of symptoms of the disease but learning to live with them in a successful way. Such expertise is described in various ways worldwide, such as experiential expertise, lay expertise, and context vs. content expertise (Attygalle, 2017; Springett et al., 2007; Williams, 2014). These terms seem to reflect subtle differences in the content of activities. For example, context expertise highlights the hierarchical organizational structures in mental health units. Experiential expertise mostly refers to relations with people with similar past experiences who have supported one’s social identity construction process. In this paper, I use the terms “expert by experience” and “experiential expertise.”

The knowledge of experts by experience and professionals is structured in different ways; nonetheless, both can be used together meaningfully. This requires a clear demarcation of the tasks that professionals are responsible for based on mandatory regulations. At the same time, experts by experience need to understand what tasks their work can extend to. In recent years, the positions of different types of knowledge and the roles of mental health experts by experience as part of mental health services have been studied intensively, especially in the field of survivor research (e.g., Brosnan & Kirby, 2016; Chassot & Mendes, 2015; Dasgupta, 2011). Experts by experience have been concluded to play an important role in reaching a better understanding of mental health problems and the ways to respond to them (Humpston, 2014; Sapiro & Ward, 2020). Experiential expertise has also been studied by analyzing the relations between peer activities, work of experts by experience, and professional knowledge (Beresford & Boxall, 2015). The construction of the social identity of mental health experts by experience has previously attracted little attention (Ramon, 2018). My study highlights this perspective with the aim of clarifying the construction of social identity when acting as an expert by experience.

## Social Identity

I address the importance of social roles and social identity in my research (Hall & Gay, 1996), and I highlight the fact that personal and social identities are intertwined with each other and develop mainly in the context of social interaction (Goffman, 1990; Harré, 1983). As an essential part of social identity, a person’s individual perception of oneself is built on a continuum formed by the personal and social identity (Tajfel, 1981; Turner, 1982). Experiences related to constructing a social identity are situational, personal, and changing (Raskin & Debany, 2018). These experiences can lead to changes in belonging to different groups and can affect the construction process (Koski-Jännes, 2002), in which individuals identify with groups whose members have partly similar backgrounds and share future expectations that they consider important. As a result, perceptions of groups close to a person’s own worldview will become particularly significant and have a substantial impact on the construction of social identity (Mead, 1967).

Bauman (1990) uses the concept of “ingroup” for groups close to a person. Previous research shows that mental health experts by experience feel a special sense of belonging to their ingroups of experts by experience (Faulkner, 2017; Dasgupta, 2011). The members of an ingroup are evaluated more positively than those of outgroups (Hannum, 2007). Goffman uses the concept of “sympathetic others” to refer to ingroups, consisting of people who share similar experiences. In an extended form, sympathetic others also include people who, through collaboration or interaction, have access to some experiences common to an ingroup (Goffman, 1990). For example, mental health professionals who work long-term alongside experts of experience can be considered as sympathetic others.

Experiencing mental health disorders can cause a sense of losing control over one’s life and previous social identity, consequently hindering one’s ability to function (Hall & Gay, 1996). A person’s profession and associated status are an important part of their social identity in modern life (Ulfsdotter Eriksson & Linde, 2014). Facing mental health disorders may interrupt one’s career or even terminate it permanently, thereby erasing one’s professional identity and chances of continuing life as it was before (see, Mental Health America, 2023). Although acting as an expert by experience is only one of the factors affecting social identity, it has a significant role to play. The meaning of former social groups connected to work, hobbies, and relatives may diminish when mental health disorders arise (Sapiro & Ward, 2020).

Although the position of a mental health expert by experience is not a profession requiring a degree, constructing the social identity and finding a new balance in life can be realized through pertinent education and acting as an expert by experience. There are no generally accepted guidelines or criteria for pertinent education for experts by experience in Finland. The education often includes both practical information about recovery and practice of practical skills. Other contents can include basic information about experiential expertise, interaction, mental health service system, self-reliance, and communication (Laitila, 2019; Sunderland & Mishkin, 2013). In Finland, the education of experts by experience takes around one year (Kivistö et al., 2023).

Constructing the identity requires adaptation to new situations, social demands, and requirements concerning one’s own competencies (Hurrelmann, 1988). This perspective is central to the present study and its emphasis on the importance of social roles and identities (Hall & Gay, 1996; Haslam et al., 2016) while considering the intertwining nature of personal and social identities (Goffman, 1990; Harré, 1983). The social identity of an expert by experience is constructed through work involving various tasks and subjective experiences, which may have a significant impact on a person’s social identity.

The construction of social identity is widely understood as an ongoing and lifelong process (e.g., Owens et al., 2010). Because mental health disorders change the person who experiences them, experiential expertise has a strong impact on the construction of social identity (Tajfel, 1981; Turner, 1982). The social identities of experts by experience are partially built in encounters with professionals representing the mental health care system (Jones et al., 2021). Professionals support the success of experts by experience in their work (e.g., Trevillion et al. (2022). This increases the opportunities for new roles for those who act as experts by experience. The information produced in my study has clear limitations. Because I have collected information from mental health professionals and experts by experience who have a long history of working together, the information concerning the attitudes of mental health professionals can be positively weighted.

This study aims to answer the following research question: What meanings do mental health experts by experience associate with their social identities? To answer this question, I consider the subjective meanings given to experiential expertise. Some may consider experiential expertise an intermediate step in moving forward in life. Others may consider it a more permanent part of their social identity. Acting as an expert by experience may change a person’s social identity from that of a patient to that of a responsible and independent actor (e.g., Brand et al., 2021). Education for experts can be built on external expectations to some extent. The content of the training can be structured to meet the goals of mental health professionals. Therefore, their social identity can be viewed as an imposed identity, the characteristics of which are not fully influenced by the experts themselves (Hannum, 2007; Davis, 2015). The education possibly produces experts by experience who adapt well to the work culture of municipal mental health work units.

## Research Material and Method

### Participants

This study involved 28 participants (age: 20–65 years), of whom 18 were experts by experience and 10 were mental health professionals. There were twenty women, one transgender person, and seven men among the participants. The experts by experience who participated in the study had careers lasting 1–20 years. Their educational and work backgrounds varied. Some did not have vocational education, and some had higher education. The professionals who participated in the study were social workers, psychiatric nurses, occupational therapists, and one doctor specializing in psychiatry. They had careers lasting 1–30 years. Most had also worked in several mental health work units.

I acquired the participating experts by experience through nongovernmental mental health associations. I have been involved in mental health organizations and have collaborated in this field nationally for 10 years. Therefore, I could easily find experts by experience as potential study participants through the help of familiar employees in organizations, compared with the effort of finding previously unknown participants. Participating experts by experience had expert by experience education meeting the criteria for participation. In addition, regular long-term work in a mental health unit was required. Specific mental health diagnoses were not asked. The experts by experience who attended the focus group discussions had completed their education through nongovernmental mental health associations. However, they did most of their work together with mental health professionals in municipal mental health units and worked only occasionally in the associations, which also supported them in their work by providing clinical supervision. All experts by experience acted as part-time employees at the time of the study. From the perspective of social identity, the participants had unique backgrounds in terms of education, previous professions, careers, and life experiences.

The mental health professionals were recruited from municipal mental health units. They had earlier experience in working together with experts by experience. Experts by experience and professionals work together, but in such a way that each brings their own special expertise to the collaboration. When working together, the attitudes of mental health professionals may affect the social identities of experts by experience (Happell et al., 2022). As “sympathetic others”, mental health professionals can support the experts by experience in their work (Goffman, 1990). In this study, I also consider the special characteristics of municipal mental health work units, established practices of such units, and positions regulated by traditional professional hierarchies that may affect the social identities of experts by experience.

### Research Material Collection

The research material consists of five focus group discussions conducted in three Finnish cities during 2015–2018. I chose focus group discussions because I aimed to achieve coproduced data and shared, cumulative meanings about experiential expertise (Hennink, 2007). Two discussions were conducted in 2015 and were attended only by experts by experience. Three discussions were conducted in November and December 2018 and were attended by both mental health experts by experience and mental health professionals.

All the discussions were structured in a similar way. In the beginning, the participants freely discussed the meanings, aspects, and experiences related to the work of experts by experience in municipal mental health units. It was important to talk freely at the beginning to achieve a confidential atmosphere. Thereafter, the groups discussed the importance of being an expert by experience for social identity. The groups discussed the importance of being an expert by experience for social identity in greater detail. The following themes were discussed in the groups: (1) personal experiences of mental health problems, (2) personal importance of belonging to a group of experts by experience, (3) meanings of working together with professionals, and (4) possible meanings assigned to the self-perception of experts by experience. I structured the themes of the discussions on the results of previous studies (e.g., Beresford & Boxall, 2015; Dasgupta, 2011; Faulkner, 2017; World Health Organization, 2021) and the knowledge accumulated from previous long-term work with experts by experience.

In the group comprising only experts by experience, the discussion was peer-supportive and emphasized the ingroup spirit (Trevillion et al., 2022; Davidson et al., 2012). In the mixed groups, the discussion emphasized the consideration of the different work tasks of experts by experience (Jones et al., 2021; Davis, 2015).

I served as the moderator and aimed to facilitate discussion. My educational and professional background supported data collection. I have a professional background as an advisor in housing services for mental health rehabilitees as well as a teacher at music colleges, high schools, and a university of applied sciences. As a teacher, I have gained experience in guiding groups; this contributed to the success of the focus group discussions. At the same time, I had to sometimes consciously avoid participating in the discussion because knowing the research topic well tempts you to contribute your own views. Altogether, the 10 h of recorded focus group discussions resulted in 90 pages of transcription. The material was in Finnish and was translated verbatim into English.

### Analytical Proceedings

In the analysis, I used a thematic design based on a material-driven approach (Braun & Clarke, 2006). The research material was sorted and labeled as a part of the qualitative research. I chose thematic analysis as an analysis method because it enables the organization, categorization, description, and interpretation of the collected research material (Braun & Clarke, 2006; Ayres, 2008). The method makes it possible to identify the similarities and differences as well as the hidden meanings in the research materials. It can be used to analyze the characteristics of the research materials and to form theoretical models (Nowell et al., 2017). Owing to my work background in mental health work, a subjective element is inherent in the analysis process. For example, Bergman and Coxon (2005) emphasize researchers’ subjectivity and context-dependent nature of categorization during the analytical process.

I started the process by examining the content of the discussions and the expressions that I thought would bear relevance to the social identity of an expert by experience. I then formed themes based on the material from the viewpoint of the research question and covered the research object as comprehensively as possible (Braun & Clarke, 2021). I divided the material into four categories through which the social identity of experts by experience is constructed and focused on the meanings of the activities that the experts associate with their social identity. I also sought to find out on whose terms the activities and social identities are defined and justified (Hannum, 2007). The units of analysis emerged from the research material, and their interpretation and categorization were guided by the theories of social identity. The focus of the interpretation was on the importance of ingroups and on the impact of social interaction on the identity of a mental health expert by experience (Goffman, 1990; Bauman, 1990; Tajfel, 1981; Turner, 1982).

### Ethics

I followed the guidelines on ethical principles issued by the Finnish National Board on Research Integrity in all stages of the research process (Finnish National Board on Research Integrity TENK, 2019). The ethical review was not required because the participating experts by experience were not clients of municipal mental health units at the time of the research. Before collecting the research material, I applied for research permits according to the official guidelines of the cities where the research was conducted. Participation was voluntary and was based on informed consent, and I respected the autonomy of the participants by allowing them to share their experiences to the extent they wanted. The participants had a right to discontinue or end their participation at all stages of the research.

I told the participants in detail what the study was about and what kind of research they were committing themselves to. Having received the research permits, I organized presentations for the participants, where I described the topic, perspective, method, and implementation of the research. During the presentations, I gave the participants a research bulletin and the consent form, which was signed and returned by those who agreed to join in. For the professionals at municipal mental health units, I sent the research bulletin and consent forms through their contact persons, to whom they also returned the signed forms. I told the participants that the focus group discussions are recorded and thereafter transcribed. To ensure that the participants could not be identified, I removed all personal information from the material during the transcription process, including names and places. Further, I removed the names of municipal mental health units, nongovernmental mental health organizations, and educational institutions. I also ensured anonymity by marking the quotations of the speakers with numbers and codes. For example, EbyE/GE refers to experts by experience in groups for experts by experience only; EbyE/MG, to experts by experience in mixed groups; and P/MG, to professionals in mixed groups.

## Results

### Changes in Social Identity Caused by Mental Health Problems

Many people can have a strong fear of losing self-control when faced with mental health disorders. The study participants reported that their mental health disorders led to a change in their social identity, which took them time to accept. Work communities as well as social networks built around leisure activities are important for people’s social identity. In a new situation, it can be difficult to experience a feeling of belonging. According to the participants, it was difficult for them to talk openly about changes in their own situation.

“I had in fact trouble with my identity when they asked me where I worked, you know, what can you say, and [Name] just told me to say that I have a job now, so I wouldn’t have to explain anything further.” (EbyE 15/GE).

Mental health disorders can also lead to subsiding or severed social relationships. A feeling of belonging to a community is important for the construction of social identity. When mental health disorders arise, it is necessary to be able to adapt to a new life situation. The participants reported that the support of experts by experience helped them to move forward and to build their identity.

I have a strong experience of having to leave my job because of depression. It was hard for me to say that I’m depressed. It was a new identity, because when working as a teacher, I was a teacher. Through this process, well, I am an expert by experience, and I am me and I really see my own identity in a different light and my whole life thereafter.” (EbyE 8/MG).

Mental health professionals understand the impact of being out of work on social identity. They nonetheless acknowledge the overemphasized importance of being employed as a measure of human dignity.

“I also constantly try to remind myself and my clients that getting a job or being employed is something that many people consider a kind of measure of human dignity. But it’s good to remember that it isn’t always realistic and it’s not the fault of the individual.” (P 17/MG).

Based on the research material, encountering mental health disorders has a significant impact on social identity, as it brings on a new life situation and forces people to thoroughly evaluate their own situation.

### Ingroup Supporting Social Identity

Mental health disorders may lead to avoidance of other people and alienation. Joining other people becomes increasingly difficult or even overwhelming. However, an ingroup can become an important factor in moving forward in life. According to the participants, getting started as an expert by experience felt relieving.

“And, so, I didn’t have the courage, I had social fears, so there was no way I could go anywhere. Right from the start, the experts by experience here fostered the kind of atmosphere that you can participate and go. Like, you met people who you could talk with. Truly a miracle, considering that I had been sort of alone for decades.” (EbyE 20/GE).

It is characteristic for people to seek out others with a similar worldview, and if one encounters mental health disorders, this behavior may be highlighted. Former social relationships and conversations may seem meaningless in a new life situation, and the participants emphasized the importance of their ingroup of experts by experience for themselves.

“When you’re among experts by experience, things are no longer so awfully superficial. As you listen to another expert by experience…you get a genuine feeling that their problems are real, not something superficial.” (EbyE 4/GE).

“At least I feel that in this group what I have known and seen you, so I feel this as an incredible feeling that how you are such great friends.” (EbyE 3/GE).

Experiences of being left alone and feelings of worthlessness are common among people with mental health disorders. Belonging to an ingroup is important and can significantly support recovery and personal well-being, as shown by the following excerpt.

“I feel that I’ve always been rejected in my life. So, in the group of experts by experience they actually listen to me, and it’s been really important. The more I tell my own story, the more I can accept myself and my own mistakes. If I have run away from them and pushed them aside before, they now become part of me.” (EbyE 2/MG).

Based on the research material, belonging to an ingroup of experts by experience has a wide-ranging effect on the construction of social identity. It enables the creation of a social network that one finds meaningful and therefore wants to commit to. In addition, it supports personal identity by enabling equal and genuine encounters with others.

### Meaning of Professionals’ Support for Social Identity

The concept of sympathetic others has been used when referring to ingroups. Sympathetic others may also include individuals who, through long-term interaction, are able to partially reach the world of experience of the ingroup. Mental health professionals and experts by experience typically work together for long periods of time in Finland. This allows professionals to gain a deeper understanding of the work of the experts by experience. The professionals who participated in the study talked about their support to experts by experience, thus opening up the importance of experiential expertise from their own perspective. Nevertheless, they emphasized that the responsibility for all care measures lies on mental health professionals. The specific characteristics of municipal mental health units and the positions dictated by the traditional professional hierarchies were clearly visible in the study. The position of an expert by experience is thus partly defined by external factors.

“I’m responsible for healthcare and that it remains at a level that is therapeutic. The sharing of experience and related knowledge is then the job of the expert by experience. I kind of think that we are after all part of the system, and we have certain responsibilities. When you make it absolutely clear, it also encourages experts by experience to tell their story, which is the most important and richest one.” (P 2/MG).

“I think there needs to be more of a confidential discussion here as well. Such a job development discussion about the roles of experts by experience between professional workers and those with experience, who do that work even as paid employees” (EbyE 4/MG).

Mental health professionals support experts by experience in many ways. Based on the research material, they consider the work of the experts significant and yet different from their own duties. The objective is to have a clear division of roles in work involving experts by experience. The experts will bring a perspective based on their own experience to work that is carried out together with professionals, who value the input.

“As an employee, I think that experts by experience give you a valuable model; I’m allowed to tell my own story. I can collect my own thoughts and past things into a story. Then again, you realize that your own story about yourself always changes when the emphasis changes. I think it’s an awesome model, to actually learn to tell the story.” (P 8/MG).

Common meanings were found in the narrations of the professionals and experts by experience who participated in the study, indicating that the professionals have reached the position of sympathetic others. The meanings given to experiential expertise seem to have been developed and shared during long-term cooperation. For experts by experience, appreciation coming from professionals is important. The experts who participated in the study spoke about their encounters with the professionals in an appreciative tone.

One professional emphasized understanding the challenges faced by experienced experts.

“We had this preparatory meeting first, and I noticed that the professionals, how they listen to you. I was completely equal there. I didn’t feel like a former patient who is there telling something, and they take a few notes, should they feel like it. Instead, we actually had a sort of, you could almost say a professional discussion there.” (EbyE 9/GE).

“I see it is very good that the experts by experience, our customers, patients, service users are involved in this operational development work. It is very valuable. It’s not like educated people think among themselves and things are then introduced based on hypotheses and interpretations.” (P 5/MG).

The importance of support from and cooperation with professionals was important. An atmosphere of appreciation toward the other party had developed between the professionals and experts by experience. People perceive their attachment to an ingroup more positively than their attachment to an outgroup. The professionals in the study had gained the trust of the experts by experience. Both parties valued each other, and the experts found the support of the professionals important.

### Individual Meanings of Social Identity

Being an expert by experience and the related social network have profound personal meanings. After experiencing mental health disorders, individuals redefine their own identity in relation to the views of others. The study participants recounted how important it was for them to act as an expert by experience.

“Each time I get a chance to speak or participate in something, this feeling of respect emerges – that I actually am worth something.” (EbyE 19/GE).

The meanings experienced are unique. Based on the research data, the unifying factor is that individuals feel they have been able to move forward in life. Their identity has changed from being a subject of mental health services to being an independent actor capable of helping others who have had the same kind of experiences.

“I think being an expert by experience was pretty important, because like others also said, it changed my view of myself, and my identity. I no longer have to be a patient.” (EbyE 10/MG).

The strengthening of independent agency through acting as an expert by experience is rooted in the fact that individuals have different goals that are associated with their individual backgrounds and personalities. Externally assigned roles for experts by experience are no longer accepted as such. The experts interact socially in their individual ways and build their identities using their own strengths.

“When I was interviewed by [name of the magazine] on mental health issues, I thought already then that I’m the kind of person who likes to perform and take part in politics. I don’t think that I have to withdraw from politics for being a transgender person. I see myself as a normal person. I try to expand the very narrow and stereotypical image of human beings. Surely, I am something more than, I’m not just a walking gender.” (EbyE 18/GE).

Working as an expert by experience involves a strong engagement in moving forward in life. The work may alleviate the weakening of life control that may be experienced because of mental health disorders. Based on the research material, acting as an expert by experience influences people’s self-perception and generates resources for solving problems:

“I am the expert on myself and my illness, or illnesses, and nobody else. And then there’s my relationship with myself and the way it constantly evolves as an expert by experience.” (EbyE 11/MG).

“Now I can live with this a lot better than before. I’m aware that the illness is not my whole identity, but a part of me only. It also helps a lot that I’m OK with myself in this, so as to deal with this illness then.” (EbyE 19/MG).

Based on the research material, working as an expert by experience bears different personal meanings. All the participating experts said that the activity had helped them move forward in life and opened up new perspectives on the future.

## Discussion

**Fig. 1:**
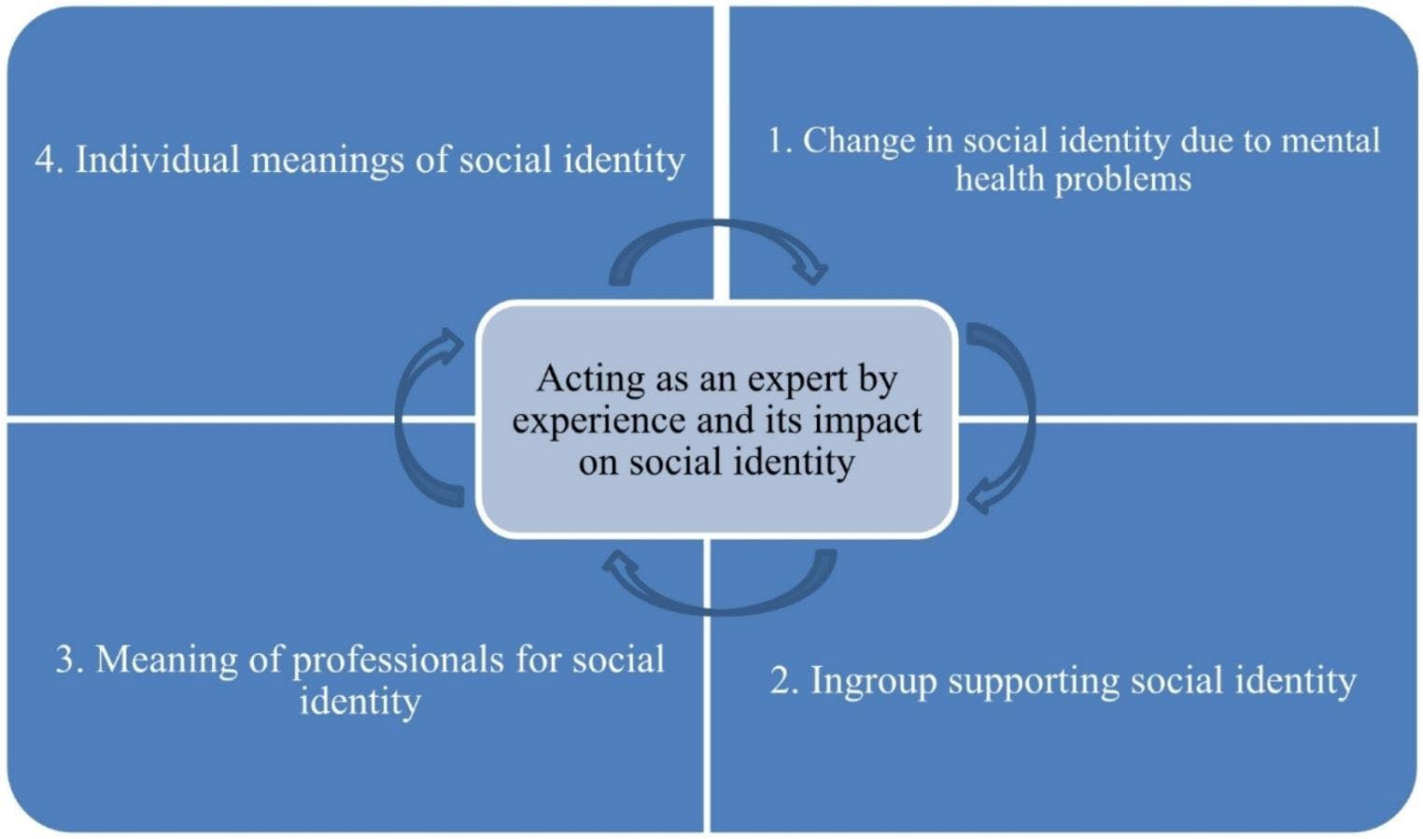
Factors constructing social identity of mental health experts by experience

In this article, I addressed the reconstruction of the social identity of experts by experience after encountering mental health disorders. I highlighted the identity-shaking effect of mental health disorders and the importance of ingroups in moving forward in life. I connected the construction of the identity to support given by professionals in mental health units through long-term co-work with the experts. The reconstruction process of social identity has clear points of convergence with development of peer provider identities (Simpson et al., 2018; Davidson et al., 2012). One of those is the recovery-oriented peer provider model, where the focus is on the process of inner growth and identity building while acting in the role of a peer (Moran, 2017). The process of building a new social identity is not linear but includes different stages. From this study, four factors are concluded as key elements that move the process forward. Figure 1 shows the four factors affecting the social identity of mental health experts by experience.

The construction of social identity requires people and relationships, as the perception of self is formed through social interaction (Goffman, 1990). Mental health disorders can lead to subsiding or severed social relationships, avoidance of other people, and alienation (Mushtaq et al., 2014; Prince et al., 2017). According to previous studies, many people have a strong fear of losing self-control when faced with mental health disorders (Hall & Gay, 1996). My study shows that facing mental health disorders actuates the process of social identity change. Belonging to an ingroup and relations with other groups are important factors in the process. The new social identity develops individually based on the person’s previous life history. All experts by experience cannot always become attached to the ingroup without difficulties, and some people may feel that they remain outsiders. In such cases, group formations can be a threat to the self and identity (Ellemers et al., 2002). After experiencing mental health disorders, individuals redefine their own identity in relation to the views of others (Tajfel, 1981; Harré, 1983). The feeling of belonging to a community is important for the construction of social identity (Tajfel, 1981), and when mental health disorders arise, it is necessary to be able to adapt to a new life situation (Hurrelmann, 1988). In the new situation, it can be difficult to experience a feeling of belonging (Prince et al., 2017).

Previous studies have shown that mental health disorders often lead to experiences of losing control over one’s life (Hall & Gay, 1996), as long-term work- or hobby-based social networks on which a person’s social identity is built may weaken or break. Impaired social relationships are common among people with mental health disorders (Prince et al., 2017). According to my research, experts by experience must consider profound questions about their identity and future in their new life situation. Joining other people becomes increasingly difficult or even overwhelming. However, an ingroup can become an important factor in moving forward in life (Simpson et al., 2018; Dasgupta, 2011). The support provided by group members builds confidence in one’s chances of success and thereby supports the development of new social identity (Puschner et al., 2019; Sapiro & Ward, 2020). Understanding the peer role and working as experts by experience are meaningful for awakening social consciousness and reconstructing the social identities of experts by experience (see, Grundman et al., 2021). By becoming an expert by experience, a person joins a social group that eventually forms an ingroup (Dasgupta, 2011; Bauman, 1990). An ingroup of experts by experience is an important social community that supports structuring one’s life situation and helps in finding a direction for the future. In the reconstruction of social identity, it is essential that those in the ingroup have more or less similar experiences of mental health disorders and a shared worldview (Mead, 1967). As an expert by experience, a person builds an understanding of oneself through reflection with the members of the ingroup (Tajfel, 1981). Compared with outgroups, an ingroup formed by experts by experience is seen in a particularly positive light (Koski-Jännes, 2002; Hannum, 2007). According to the results of my research, an ingroup makes it easier to talk about one’s own life situation and promotes a sense of belonging to a community.

Previous studies have not paid much attention to the relationships between experts by experience and professionals or to the construction of the experts’ social identity. Instead, the different roles of peer activities, experts by experience, and professionals and the relationships between the people involved have been studied extensively (Moran, 2017; Beresford & Boxall, 2015; Davidson et al., 2012). Based on the results of my research, experts by experience value the support given by professionals and find it an important source of self-confidence and courage to act in the expert position. Therefore, support from professionals in social interaction is an important contributor to the success of the work of experts by experience and to their social identity (Ramon, 2018). The positions of experts by experience can be partly defined by external factors (Hannum, 2007). The experts by experience seek individual solutions to identity construction and find the roles offered to them somewhat narrow and restrictive (Davis, 2015). Based on the research results, the individual meanings of acting as an expert by experience are considered particularly important. Being an expert by experience helps the person to move forward in life and to find individual ways to construct social identity. It can also be a driving force in creating and supporting social relationships and mental well-being.

In conclusion, the successes experienced in the work as an expert by experience can create a foundation for future dreams (Mental Health America, 2023; Davis, 2015; Haslam et al., 2016). In the long run, it can also help one to return to work or studies, thereby giving life a new purpose and positive meanings. Taking this into account in the future when planning training and assignments for experts by experience could benefit the experts and their clients and enhance the effectiveness of mental health services. In future studies, it would be important to investigate how the work of experts by experience could be designed on a more individual basis by considering people’s unique backgrounds and special skills. This study calls attention to the meaning of acting as an expert by experience and to its impact on the social identity of the experts within the specific work culture of municipal mental health care units. My study and focus groups as a used method has clear limitations. In a group, sharing sensitive personal matters can be difficult. The data is quite small as a whole and was collected in municipal mental health units, in which the unique work culture can impact perceptions of experts by experience. Nonetheless, the data reveals important elements of the subject.
